# A Mutation in the Herpes Simplex Virus Type 1 (HSV-1) UL29 Gene is Associated with Anti-Herpesvirus Drugs’ Susceptibility

**DOI:** 10.3390/v16121813

**Published:** 2024-11-21

**Authors:** Souichi Yamada, Shizuko Harada, Hikaru Fujii, Hitomi Kinoshita, Phu Hoang Anh Nguyen, Miho Shibamura, Tomoki Yoshikawa, Madoka Kawahara, Hideki Ebihara, Masayuki Saijo, Shuetsu Fukushi

**Affiliations:** 1 Department of Virology 1, National Institute of Infectious Diseases, Tokyo 162-8640, Japan; shizuko@niid.go.jp (S.H.); knsht@niid.go.jp (H.K.); nguyenanh@niid.go.jp (P.H.A.N.); ytomoki@niid.go.jp (T.Y.); kawahara@niid.go.jp (M.K.); hebihara@niid.go.jp (H.E.); msaijo@niid.go.jp (M.S.); fukushi@niid.go.jp (S.F.); 2 The Faculty of Veterinary Medicine, Okayama University of Science, Imabari 794-8555, Ehime, Japan; hikar-fujii@ous.ac.jp; 3 Center for Surveillance, Immunization and Epidemiologic Research, National Institute of Infectious Diseases, Tokyo 162-8640, Japan; miho-s@niid.go.jp; 4 Health and Welfare Bureau, Sapporo City 060-8611, Hokkaido, Japan

**Keywords:** herpes simplex virus type 1 (HSV-1), UL29, acyclovir, drug resistance

## Abstract

Herpes simplex virus type 1 (HSV-1) acyclovir (ACV) resistance is acquired by mutations in the viral thymidine kinase (TK) or DNA polymerase (DNApol) genes. We previously obtained an ACV-resistant clone (HSV-1_VZV_TK_clone α) by sequential passages of HSV-1_VZV-TK, a recombinant virus which lacked its endogenous TK activity and instead expressed the varicella-zoster virus (VZV) TK ectopically. HSV-1_VZV_TK_clone α had been generated using an HSV-1_BAC in the presence of increasing concentrations of ACV. The ACV-resistant clone bore normal TK and DNApol genes. Here, we deployed next-generation full-genome sequencing of HSV-1_VZV_TK_clone α and identified a single nucleotide substitution, resulting in a P597L missense mutation in the UL29 gene product, the ICP8 protein. Recombinant HSV-1 encoding a P597L ICP8 protein was generated, and its properties and ability to confer drug resistance were analyzed. No difference in virus growth and UL29 expression was observed between the mutant recombinant, the wild type, and a revertant mutant viral strain, and susceptibility tests of these strains to ACV and other drugs using Vero, HEL, and ARPE19 cells identified that the recombinant UL29 mutant virus was resistant only to ACV. These results indicate that ICP8 may be involved in the anti-herpesvirus drugs’ mechanism of action on HSV-1.

## 1. Introduction

Antiviral-drug-resistant herpesvirus infections often become a major concern in the treatment of immunocompromised patients, such as those with acquired immune deficiency syndrome or organ/hematopoietic stem cell transplant recipients [1,2]. Patients with herpesvirus infections undergoing long-term antiviral drug administration have an increased risk of drug resistance [3,4,5], with the prevalence of drug-resistant HSV-1 estimated to be 4.1–7.1% in immunocompromised patients [6]. Nucleoside, nucleotide, and pyrophosphate analogs are widely used to treat patients with herpesvirus infections. The nucleoside analogs, acyclovir (ACV), penciclovir (PCV), and thymine-arabinoside (Ara-T), are phosphorylated by viral thymidine kinase (TK) to acquire their monophosphate forms. In contrast, brivudine (BVDU) is phosphorylated by viral TK into both mono- and diphosphate forms. These are then further phosphorylated by cellular enzymes, ultimately converting them into their active triphosphate forms [7,8]. ACV triphosphate is incorporated into viral DNA, resulting in the termination of viral DNA chain elongation and the inhibition of viral replication [9]. Thus, ACV-resistant herpesviruses are either viral TK- and/or DNA polymerase- (DNApol-) mutants [10,11], although the former occur more frequently than the latter [12,13]. Unlike ACV, which relies on viral TK for activation, pyrophosphate analog foscarnet (FOS) directly inhibits DNApol. The nucleotide analog cidofovir (CDV) undergoes two stages of phosphorylation by cellular kinase to be converted into its active form, CDV-diphosphate [14]. Thus, FOS- or CDV-resistant herpesviruses are generated by mutations in the DNApol gene [15,16]. Other viral proteins related to HSV-1 DNA replication, such as the single-stranded DNA binding protein ICP8 (encoded by UL29), origin binding protein (encoded by UL9), helicase–primase complex (encoded by UL5, UL8, and UL52), and processivity factor (encoded by UL42), work together during virus replication [17]. However, no reports to date have implicated these proteins in ACV activation or associated them with ACV resistance.

Recently, we reported the identification of a recombinant HSV-1 clone (HSV-1_VZV_TK_ clone α), which showed resistance to ACV despite bearing no mutations in either the viral TK and DNApol genes [18]. Here, we used next-generation sequencing (NGS), identified a mutation in the UL29 gene of HSV-1_VZV_TK_ clone α, and showed that it caused ACV resistance by analyzing recombinant viruses. This indicates that ICP8 interferes with the ACV mechanism of action in HSV-1-infected cells.

## 2. Materials and Methods

### 2.1. Cells and Viruses

Vero cells were cultured in Dulbecco’s Modified Eagle Essential Medium (DMEM; FUJIFILM Wako Chemicals, Richmond, VA, USA) supplemented with 5% fetal bovine serum (FBS; Biofill Australia Pty., Melbourne, Victoria, Australia), 100 U/mL of penicillin, and 100 µg/mL of streptomycin (ThermoFisher Scientific K.K., Tokyo, Japan). Human retinal pigment epithelial (ARPE19) cells were cultured in DMEM/Ham’s F-12 medium (FUJIFILM Wako Chemicals, Richmond, VA, USA), supplemented with 10% FBS, 100 U/mL of penicillin, and 100 µg/mL of streptomycin (ThermoFisher Scientific K.K., Tokyo, Japan). Human embryonic lung fibroblast (HEL) cells were cultured in Eagle’s minimal essential medium (FUJIFILM Wako Chemicals, Richmond, VA, USA), supplemented with 10% FBS, 100 U/mL of penicillin, and 100 µg/mL of streptomycin (ThermoFisher Scientific K.K., Tokyo, Japan). Each recombinant HSV-1 was propagated and titrated in Vero cells, as described previously [19].

### 2.2. Sequencing

An NGS sample was extracted from HSV-1_VZV_TK_clone α-infected Vero cells using a DNA mini kit (QIAGEN, Hilden, Germany). NGS was performed using an Ion PGM System (ThermoFisher Scientific K.K., Tokyo, Japan), Ion 314 v2 tips, and alignment and analysis of variation using CLC Genomic Workbench software (QIAGEN, Hilden, Germany). The BigDye Terminator Cycle Sequencing Kit ver. 3.3 (PE Applied Biosystems, Waltham, MA, USA) was used for Sanger sequencing reactions, and the reactions were analyzed using the ABI 3500 xL Genetic Analyzer (PE Applied Biosystems, Waltham, MA, USA). Data were aligned and analyzed using DNADynamo ver.1.616 (Blue Tractor Software, Sealand, UK). The UL29 gene sequence of HSV-1_VZV_TK_clone α strain was submitted to the GenBank database accessed on 23 August 2024 (accession no. LC833868).

### 2.3. Construction of Recombinant Viruses

A recombinant HSV-1_VZV_TK_UL29mut virus, which bore a substitution in the UL29 gene encoding a P597L missense mutation, was produced by a two-step Red-mediated mutagenesis method using a zeomycin resistance gene (introduced by pUC-Zeo) [20] with the *E. coli* strain GS1783 [17] containing the full-length infectious HSV-1_VZV_TK plasmid [18].

To generate recombinant HSV-1_BAC_UL29mut and HSV-1_BAC_UL29mut_rev viruses using the two-step Red-mediated mutagenesis procedure, we used a kanamycin resistance gene (rKAN) (introduced by pcDNA3-KanS) [21] with the *E. coli* GS1783 strain containing a full-length infectious HSV-1 F BAC (HSV-1_BAC) plasmid [22]. The plasmid was kindly provided by Dr. Y. Kawaguchi of the University of Tokyo, Japan, with permission for use from Dr. G.A. Smith of Northwestern University, IL, USA, and Dr. N. Osterrieder of Freie Universität, Berlin, Germany [20,21]. The primers are shown in Supplementary Table S1.

### 2.4. Detection of TK and ICP8 Proteins

Western blotting was performed, as described previously [19]. Briefly, Vero cells were infected with the generated recombinant viruses at a multiplicity of infection (MOI) of three per cell and harvested at 24 h post infection. The cell lysates were fractionated using sodium dodecyl sulfate (SDS)-polyacrylamide gel electrophoresis and subjected to Western blotting. To detect the HSV-1 UL29, TK, and VZV TK proteins, the membrane was allowed to react with the mouse anti-HSV-1 ICP8 monoclonal antibody (11E2, ab20194, Abcam, Cambridge, UK), rabbit anti-HSV-1 TK serum [3], and goat anti-VZV TK polyclonal antibody (sc-17554; Santa Cruz Biotechnology, Dallas, TX, USA), followed by incubation with the horseradish peroxidase (HRP)-conjugated goat anti-mouse IgG (H + L) antibody (Thermofisher Scientific K.K. Tokyo, Japan), swine anti-rabbit immunoglobulins polyclonal antibody (Dako, Santa Clara, CA, USA), and rabbit anti-goat immunoglobulins polyclonal antibody (Dako, Santa Clara, CA, USA), respectively.

### 2.5. Plaque Titration

Vero cells were infected with 10-fold serially diluted virus solutions. After a 1 h absorption at 37 ℃ in the presence of 5% CO_2_, the cells were washed with phosphate buffered saline solution, and cultured in DMEM supplemented with 2% FBS, 100 U/mL of penicillin, 100 µg/mL of streptomycin, and 0.16 mg/mL of gamma globulin (Sigma-Aldrich, St. Louis, MO, USA). Cells were harvested at designated time points, and viral infectious doses were measured using standard plaque assays in Vero cells.

### 2.6. Antiviral Drug Compounds

ACV, PCV, Ara-T, BVDU, and FOS, purchased from Tokyo Chemical Industry (Tokyo, Japan), and CDV, formally known as (S)-1-(3-hydroxy-2-phosphonylmethoxypropyl) cytosine, purchased from Cayman Chemical Company (Ann Arbor, MI, USA), were used.

### 2.7. Drug Susceptibility Test

The susceptibility of recombinant HSV-1 clones to antiviral drugs was assayed using a 50% plaque-reduction assay in Vero, HEL, and ARPE19 cells, as described previously [23]. Briefly, Vero, HEL, and ARPE19 cells were seeded in 24-well plates and infected with 50 plaque forming units of recombinant HSV-1 clones/well. After a 1 h incubation, the cells were cultured in maintenance medium, DMEM, or EMEM with FBS containing gamma globulin (Sigma-Aldrich, St. Louis, MO, USA), with the designated concentration of ACV, PCV, Ara-T, BVDU, CDV, or FOS. The 50% inhibitory concentration (IC_50_) of each antiviral compound was calculated as the concentration at which the plaque number decreased to half of that observed in a medium without antiviral agents.

### 2.8. Statistical Analysis

Statistical differences of the IC_50_ values between recombinant viruses were evaluated using ordinary (not repeated measures) analysis of variance with Bonferroni correction. Statistical significance was set at *p* < 0.05.

## 3. Results

### 3.1. Genomic Sequence Analysis of the HSV-1_VZV_TK_ Clone α Strain

**Figure 1:**
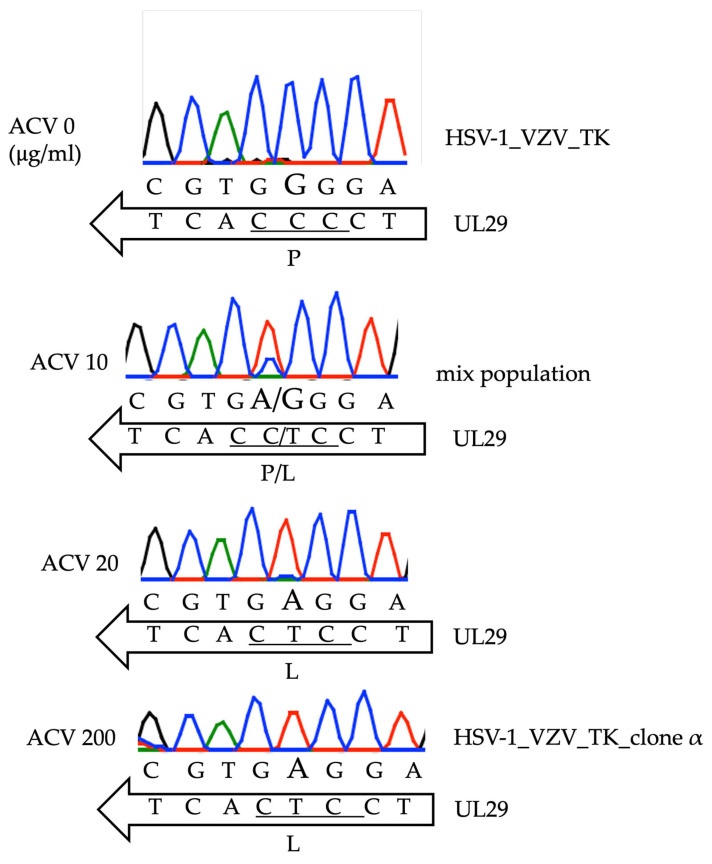
Nucleotide substitution in HSV-1_VZV_TK_clone α strain UL29 gene. The original recombinant virus (HSV-1_VZV_TK) was passed through increasing concentrations of ACV (0 μg/mL, 10 μg/mL, 20 μg/mL, and 200 μg/mL). The UL29 gene sequence was determined by Sanger sequencing of viral DNA, cultured in the presence of the specified ACV concentrations. Bold type indicates the substituted base.

We previously obtained the HSV-1_VZV_TK_clone α strain by serial passages of HSV-1_VZV_TK, in which part of the HSV-1 TK gene was replaced by the rKAN gene and the VZV TK gene was inserted in the UL50-51 intergenic region, in the presence of the increasing concentration of ACV (Supplementary Figure S1a) [18]. Initially, we used NGS to determine the whole genome sequence of the HSV-1_VZV_TK_clone α strain, except for its terminal-repeat region. The genome sequence of the HSV-1_VZV_TK_clone α strain was compared with those of the ACV-sensitive parental HSV-1_BAC strain (accession number: OK030826.1) and the original HSV-1 F strain (accession number: GU734771.1). There was a nucleotide substitution at position nt.60180 in the HSV-1_VZV_TK_clone α strain, resulting in P597L amino acid substitution in the UL29 gene as compared with the HSV-1 BAC and HSV-1 F sequences (Table 1). There were 16 nucleotide differences between HSV-1_VZV_TK_clone α strain and HSV-1 F; however, they were originated from the parental HSV-1_BAC virus genome, except for the P597L amino acid substitution in the UL29 gene (Table 1). We also identified a different number of repeat sequences in the intergenic region between the US9 and US10 genes. The nt1059 substitution in UL29 was also confirmed by Sanger sequencing. The original G nucleotide at position 1059 in UL29 was gradually replaced by A in the majority of the viral population upon ACV treatment (Figure 1).

### 3.2. ACV Susceptibility of HSV-1_VZV_TK_UL29mut Bearing a P597L Substitution

**Figure 2:**
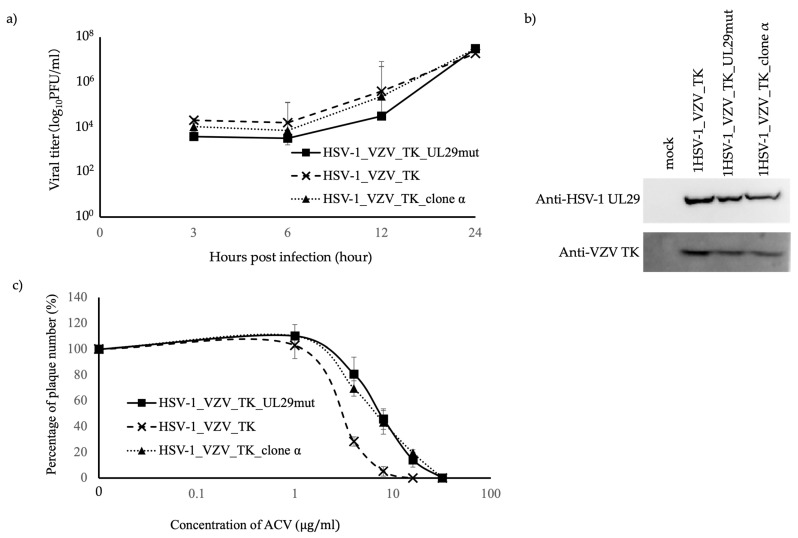
Growth properties, TK and UL29 protein expression, and ACV susceptibility of HSV-1_VZV_TK_UL29mut, HSV-1_VZV_TK, and HSV-1_VZV_TK_ clone α strain (**a**) Vero cells in 24-well plates were infected with HSV-1_VZV_TK_UL29mut, HSV-1_VZV_TK, or HSV-1_VZV_TK_ clone α strains at a multiplicity of infection (MOI) of 3, and culture supernatants and cells were recovered and frozen at the indicated times after infection. The viral titers were determined by measuring the plaque-forming units. Means and standard deviations of the virus stocks prepared from wells in triplicate were plotted for HSV-1_VZV_TK_UL29mut (closed squares), HSV-1_VZV_TK (×), and HSV-1_VZV_TK_clone α strain (closed triangles). (**b**) Expression of the TK and UL29 proteins in Vero cells infected with HSV-1_VZV_TK_UL29mut, HSV-1_VZV_TK, HSV-1_VZV_TK_clone α strain, and mock at an MOI of 3, as assessed using western blot analysis. (**c**) Sensitivity of each viral strain to ACV was determined using a plaque reduction assay on Vero cells. The replication curves of each virus in the presence of the designated concentration of antiviral drugs are shown. Each concentration was tested in triplicate, and the experiments were independently repeated three times. The results are shown as means and SDs of three experiments.

To examine whether the P597L substitution in ICP8 affected the susceptibility of HSV-1 to ACV, we introduced the corresponding nt1059 substitution in the HSV-1_VZV_TK strain using RedE/T recombination (HSV-1_VZV_TK_UL29mut) (Supplementary Figure S1b). We established that the HSV-1_VZV_TK_UL29mut, HSV-1_VZV_TK, and HSV-1_VZV_TK_clone α strains showed similar growth kinetics in Vero cells, which expressed TK and ICP8 proteins at similar levels (Figure 2a,b). We then analyzed the susceptibility of these viruses to ACV in Vero cells and found that the IC_50_ values (mean ± SD) of ACV against HSV-1_VZV_TK_UL29mut (7.2 ± 1.7 μg/mL) and HSV-1_VZV_TK_clone α (7.2 ± 1.5 μg/mL) were significantly higher than those of HSV-1_VZV_TK (2.6 ± 0.1 μg/mL) (*p* < 0.05) (Figure 2c, Supplementary Table S2).

### 3.3. Susceptibility of HSV-1_BAC_UL29mut to ACV and Other Drugs

**Figure 3:**
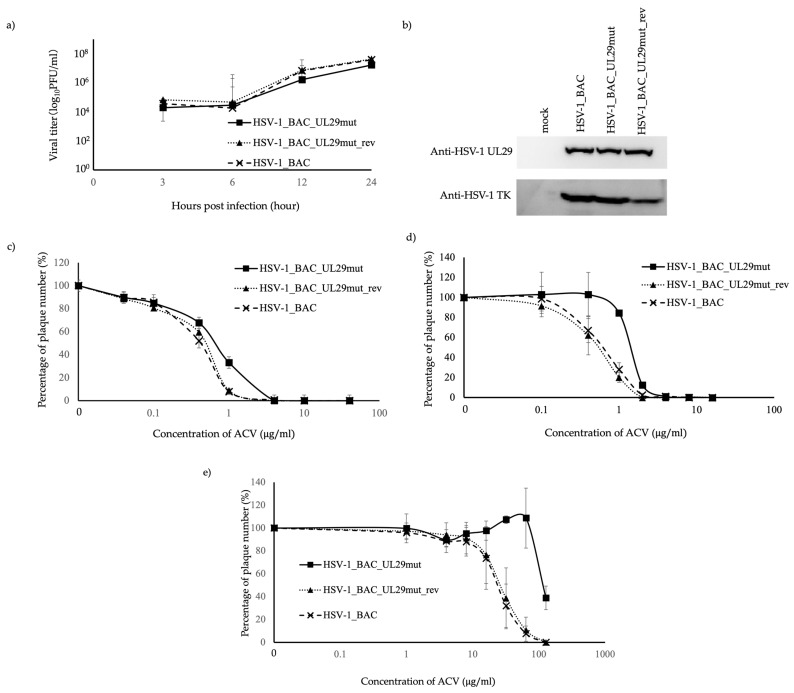
Growth properties, TK and ICP8 protein levels, and susceptibility of HSV-1_BAC_UL29mut, HSV-1_BAC_UL29mut_rev, and HSV-1_BAC to ACV. (**a**) Vero cells were infected in 24-well plates with HSV-1_BAC_UL29mut, HSV-1_BAC_UL29mut_rev, and HSV-1_BAC at a multiplicity of infection (MOI) of 3, and culture supernatants and cells were recovered and frozen at the indicated times after infection. The viral titers were determined by measuring the plaque forming units. Means and standard deviations of the viral stocks prepared from wells in triplicate are plotted for HSV-1_BAC_UL29mut (closed squares), HSV-1_BAC_UL29mut_rev (closed triangles), and HSV-1_BAC (×). (**b**) TK and ICP8 protein levels in Vero cells infected with HSV-1_BAC_UL29mut, HSV-1_BAC_UL29mut_rev, HSV-1_BAC, and mock at an MOI of 3, as assessed using western blot analysis. (**c**–**e**) Sensitivity of each viral strain to ACV was determined using a plaque reduction assay on Vero, HEL, and ARPE19 cells. The replication curves of each virus in the presence of the designated concentrations of antiviral drugs are shown. Each concentration was tested in triplicate, and the experiments were independently repeated three times. The results are shown as means and standard deviations of three independent experiments.

A mutant HSV-1 BAC, bearing a P597L missense mutation in ICP8 (HSV-1_BAC_UL29mut), was constructed from a parental HSV-1_BAC (Supplementary Figure S1c). HSV-1_BAC_UL29mut, a revertant of HSV-1_BAC_UL29mut (HSV-1_BAC_UL29mut_rev), and HSV-1_BAC showed similar growth and TK expression properties (Figure 3a,b). When an ACV susceptibility test was performed using Vero cells, we observed that HSV-1_BAC_UL29mut was susceptible to ACV (IC_50_ value of 0.65 ± 0.09) (Figure 3c). However, HSV-1_BAC_UL29mut (1.39 ± 0.02 μg/mL) showed a significantly lower susceptibility to ACV (ACV resistance) than the HSV-1_BAC (0.60 ± 0.14 μg/mL) and HSV-1_BAC_UL29mut_rev (0.45 ± 0.21 μg/mL) strains in HEL cells (*p* < 0.01). In addition, using ARPE19 cells, a significantly lower susceptibility to ACV against HSV-1_BAC_UL29mut (113 ± 1.26 μg/mL), compared with HSV-1_BAC (24.7 ± 11.0 μg/mL) and HSV-1_BAC_UL29mut_rev (27.9 ± 9.20 μg/mL) viruses, was observed (*p* < 0.01) (Figure 3d,e). Next, we examined the susceptibility to other drugs using Vero and HEL cells. We observed that HSV-1_BAC_UL29mut showed similar susceptibility to PCV, Ara-T, BVDU, and CDV as HSV-1_BAC and HSV-1_BAC_UL29mut_rev. However, the FOS IC_50_ values against HSV-1_BAC_UL29mut using Vero and HEL cells (12.0 ± 0.45 μg/mL and 28.2 ± 1.61 μg/mL, respectively) were estimated to be 50% of those against HSV-1_BAC (22.7 ± 1.91 μg/mL and 51.5 ± 9.14 μg/mL, respectively) and HSV-1_BAC_UL29mut_rev (21.3 ± 0.47 μg/mL and 48.6 ± 5.43 μg/mL, respectively), indicating that HSV-1_BAC_UL29mut was significantly more sensitive to FOS than HSV-1_BAC and HSV-1_BAC_UL29mut_rev (*p* < 0.01) (Table 2, Supplementary Table S2).

## 4. Discussion

In the present study, we have demonstrated an association between UL29 gene mutations and HSV-1 drug resistance using a recombinant HSV-1 backbone.

We detected a mutation in the UL29 gene by NGS in HSV-1_VZV_TK_clone α that showed ACV resistance, and sanger sequencing retrospectively confirmed that resistant viruses carrying the identified P597L mutation in ICP8 gradually became the predominant population in the presence of ACV. The precise proportion (G versus A) of mutation in ICP8 during viral passage in the presence of 0, 10, and 20 μg/mL of ACV remained unclear since we did not perform NGS for the clones recovered at each stage. At least, the data shown in Table 1 indicated that HSV-1_VZV_TK_clone α had G1059A substitution with 100% frequency.

HSV-1_VZV_TK_clone α and HSV-1_VZV_TK_UL29mut showed reduced susceptibility to ACV in Vero cells. However, HSV-1_VZV_TK_clone a was selected under pressure of ACV, and was derived from HSV-1_VZV_TK, which had a VZV TK but not an HSV-1 TK gene. The sensitivity of VZV to ACV is about 100 times lower than that of HSV-1 [24]; therefore, the combination of VZV TK and the UL29 mutation may have conferred higher resistance than the UL29 mutation alone. Therefore, we constructed the recombinant HSV-1 which had only a L529P mutation in the UL29 gene based on HSV-1_BAC, and then analyzed its drug susceptibility.

Nevertheless, HSV-1_BAC_UL29mut (Figure 3) did not show clear resistance to ACV using Vero cells; it showed apparent resistance using HEL and ARPE19 cells. The fact that HSV-1_BAC_UL29mut had decreased susceptibility to ACV compared to HSV-1_BAC suggests that UL29mut is associated with drug sensitivity. HSV-1 strains carrying mutations in TK have been demonstrated to show various degrees of susceptibility to ACV, Ara-T, IDU, and BV-araU among different cell types [25], and individual HSV-1 clinical strains display different infection characteristics according to cell type [26]. The UL29 gene interacts with a variety of host factors. Therefore, it is possible that the UL29 mutation shown in this study may have affected drug sensitivity through direct or indirect interactions with host factors. HSV-1_BAC_UL29mut showed no clear change in sensitivity to ACV in Vero cells, but about a 3-fold decrease in sensitivity in HEL cells and about a 4-fold decrease in sensitivity in ARPE19 cells, whereas FOS showed an approximately 2-fold increase in sensitivity in both Vero and HEL cells. The altered sensitivity of FOS, which directly affects DNApol, suggests that the UL29 mutated region alters drug sensitivity by directly or indirectly affecting UL30.

The UL29 gene encodes the multifunctional ICP8 protein, one of the most abundant early viral proteins with ssDNA binding activity [27], involved in viral genome replication, homologous recombination through complex formation with UL12 [28], and late gene expression [29]. The P597L mutation resides in the β22 strand of a β-sheet, whose β18–22 strands are involved in interactions with N-terminal strand residues [30]. However, no information regarding the association between anti-herpesvirus drug activity or resistance and this amino acid region has been established. Although ICP8 function has not been fully elucidated, ICP8 has been known to interact directly or indirectly with several viral proteins, such as UL9, a helicase, and many host proteins which function in viral genome replication [31]. Additionally, it contributes to the formation of replication compartments [32]. Therefore, ICP8 may have additional unknown functions related to DNA replication. The mechanism behind how the P597L substitution in ICP8 affects the ACV susceptibility of HSV-1 has been not uncovered. Since residue 597 is included within the DNA binding region of ICP8 [33,34], the DNA binding activity of ICP8 might be affected by the P597L substitution. A plausible explanation for the mechanism behind the ACV resistance of HSV-1 carrying mutant ICP8 might be the functional interaction of ICP8 and the HSV-1 DNA polymerase-UL42 complex [35]. There is a possibility that ICP8 involves the HSV-1 DNA polymerase-UL42 complex interacting with incoming nucleotide or antivirals [36] upon binding to single-stranded DNA, and that mutant ICP8 affects their structural conformation. Another possibility is that, since the ICP8 modulates deoxynucleotide triphosphate incorporation by HSV-1 DNA polymerase [35], the ICP8 P597L amino acid substitution could alter ACV triphosphate incorporation. Detailed analyses of the effect of amino acid substitution in ICP8 on DNA binding and/or deoxynucleotide triphosphate incorporation by using purified ICP8, DNA polymerase, and other viral DNA replication machineries are required to clarify these possibilities. In this study, the UL29 mutation did not affect viral replication and ICP8 protein production, suggesting that the mutation resides in a region that does not affect or is not involved in viral replication. However, we did not identify how this single nucleotide substitution confers drug resistance in ICP8.

The oriL, known as the initiation site of HSV-1 genome replication [37], is deleted from the HSV-1_BAC virus when propagating BAC clones in E.coli. However, there is no direct regulatory link between oriL and the transcription or translation of UL29 and UL30 genes. Alterations in oriL would not change the expression levels of these two genes and virus growth in Vero, MEF, and dTGs cells, as their regulation is independent of the nearby origin of replication [38,39]. We suggest that the deletion of oriL had no impact on ACV resistance, because the HSV-1_BAC virus without the oriL sequence was actually susceptible to ACV in Vero, HEL, and ARPE19 cells. The parental HSV-1_BAC virus has already had mutations in genes related to the genome replication process, such as ICP6. There is a possibility that the mutation on ICP6 affects ribonucleotide reductase activity and thus it is correlated with the ACV sensitivity of HSV-1 in infected cells. In fact, the inhibition of ribonucleotide reductase activity using drug compound A723U leads to the reduction in deoxynucleotide pool sizes and increased levels of ACV triphosphate in infected cells [40]. However, in this study, the HSV-1_BAC virus was shown to be sensitive to ACV. It remains unclear whether the combination of the UL 29 mutation with an additional mutation on genes responsible for the viral replication process leads to ACV resistance. It will be clarified in future studies by creating a double mutant virus. No additional mutations were found except for the terminal repeat, the sequence of which could not be determined, and the intergenic region between the US9 and US10 genes, which also constituted a repetitive sequence. These regions have not been reported to be involved in drug resistance.

The ICP8 P597L mutation resulted in the effect of ACV and FOS susceptibility (Table 2). UL29 mutant variants were also susceptible to Ara-T, BVDU, and PCV, which have the same mechanism of action as ACV. It is well known that mutations in the TK (PK) or DNApol genes that confer resistance to some drugs may not affect susceptibility to other drugs [41,42,43], and this phenomenon may also hold true for UL29 mutations. However, it remains possible that mutations in other regions of the UL29 gene could provide resistance to other drugs. The effects of HSV-1_BAC-UL29mut on other drugs, including ganciclovir, are of great interest, and we will need to analyze the effects of other drugs.

In this study, we showed for the first time that a mutation in a gene other than TK and DNApol, namely in UL29, causes ACV resistance. To clarify the clinically relevant effect of this mutation on drug resistance, it will be necessary to examine its presence or absence in patients with drug-resistant HSV-1 infections.

## Figures and Tables

**Table 1:** Nucleic acid substitution with amino acid changes in HSV-1_VZV_TK_clone α strain genes.

Gene	Gene Function	Position *	HSV-1_VZV_TK_Clone α	HSV-1_BAC	HSV-1 F	a.a Change	Frequency	Quality
UL13	protein kinase	26,904	T	T	C	C507Y	100	776.4
UL29	ICP8	60,180	A	G	G	P597L **	100	555.8
		61,007	A	A	G	NC	100	755.9
UL34	ER modeling	69,681	A	A	G	S45N	98.1	462.7
UL36	tegument protein	71,717	T	T	C	M2434V	100	73.8
		71,723	C	C	T	NC	100	102.0
		73,069	C	C	T	NC	100	384.7
UL38	capsid protein	84,529	C	C	T	C35R	100	961.3
UL39	ICP6	87,759	T	T	C	A474V	100	618.4
		88,762	T	T	C	NC	98.4	559.4
UL48	VP16	103,969	A	A	G	T212A	100	820.1
		104,340	C	C	T	NC	100	865.1
UL50	dUTPase	107,306	A	A	C	P134T	96.1	403.2
UL55	non-structural protein	115,491	A	A	G	M35I	100	423.7
UL56	structural protein	116,406	T	T	C	A137T	100	401.9
US2	tegument protein	134,690	A	A	G	L34F	97.1	304.5

* HSV-1 F strain genome position (Accession number: GU734771.1). ** Bold type was shown amino acid change from parental HSV-1_BAC strain. NC: not changed.

**Table 2:** ACV, BVDU, Ara-T, PCV, CDV, and FOS susceptibility of HSV-1_BAC_UL29mut, HSV-1_BAC_UL29mut_rev, and HSV-1_BAC strains.

Cells	Viruses	IC_50_ (Mean ±SD μg/mL)					
		ACV	BVDU	Ara-T	PCV	CDV	FOS
Vero cells	HSV-1_BAC_UL29mut	0.65 ± 0.09	0.75 ± 0.13	4.67 ± 0.51	0.51 ± 0.06	0.45 ± 0.03	12.0 ± 0.45 *
	HSV-1_BAC_UL29mut_rev	0.47 ± 0.03	1.35 ± 0.13	4.37 ± 0.82	0.56 ± 0.02	0.32 ± 0.04	21.3 ± 0.47
	HSV-1_BAC	0.41 ± 0.06	1.15 ± 0.25	4.29 ± 0.01	0.56 ± 0.03	0.47 ± 0.04	22.7 ± 1.91
HEL cells	HSV-1_BAC_UL29mut	1.39 ± 0.02 *	0.11 ± 0.01	0.42 ± 0.15	1.38 ± 0.44	0.52 ± 0.18	28.2 ± 1.61 *
	HSV-1_BAC_UL29mut_rev	0.45 ± 0.21	0.19 ± 0.04	0.66 ± 0.17	1.77 ± 0.20	0.33 ± 0.03	48.6 ± 5.43
	HSV-1_BAC	0.60 ± 0.14	0.14 ± 0.02	0.68 ± 0.08	1.59 ± 0.11	0.44 ± 0.04	51.5 ± 9.14
ARPE19 cells	HSV-1_BAC_UL29mut	113 ± 1.26 *	NT	NT	NT	NT	NT
	HSV-1_BAC_UL29mut_rev	27.9 ± 9.20	NT	NT	NT	NT	NT
	HSV-1_BAC	24.7 ± 11.0	NT	NT	NT	NT	NT

NT, not tested; *, significantly different from HSV-1_BAC_UL29mut_rev and HSV-1_BAC (*p* < 0.01).

## Data Availability

The data presented in this study are openly available in the GenBank database by accession no. LC833868.

## Supplementary Materials

The following supporting information can be downloaded at: https://www.mdpi.com/article/10.3390/v16121813/s1: Figure S1, structure of HSV-1_VZV_TK, HSV-1_VZV_TK_UL29mut and HSV-1_BAC_UL29mut; Table S1, primers used for mutagenesis; Table S2: ACV susceptibility of HSV-1_VZV_TK_UL29mut, HSV-1_VZV_TK, and HSV-1_VZV_TK_clone α strain.
