# Supplementary material for: A Mutation in the Herpes Simplex Virus Type 1 (HSV-1) UL29 Gene is Associated with Anti-Herpesvirus Drugs’ Susceptibility
Source: Viruses. 2024 Nov 21;16(12):1813. doi: 10.3390/v16121813 (PMC11680290; doi:10.3390/v16121813)
Supplement: Supplementary file 1 [file viruses-16-01813-s001.zip › viruses-3206577-supplementary.pdf]

Supplemental Materials

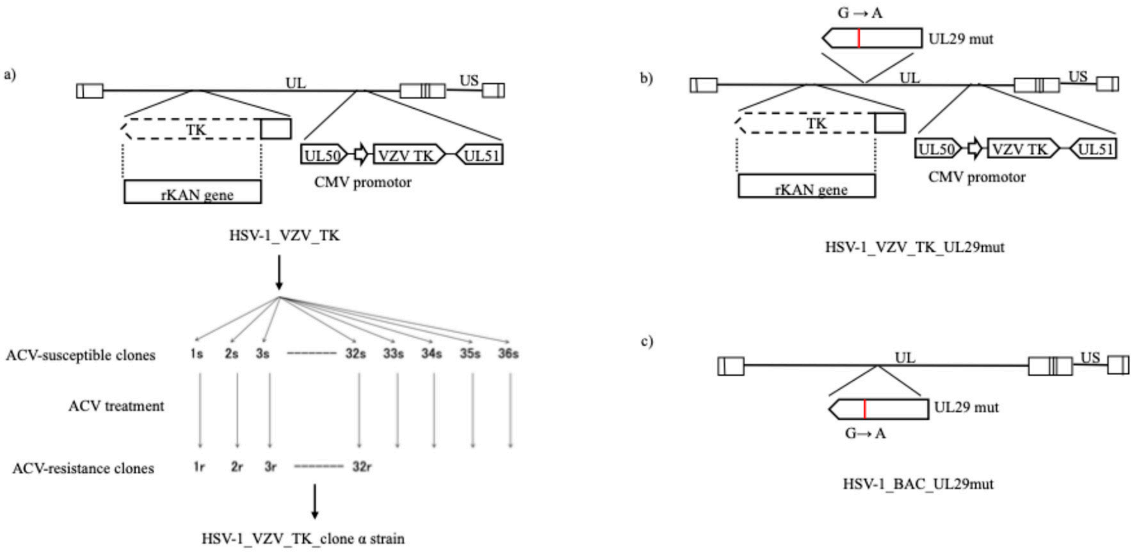

Supplemental Figure S1. Structure of HSV-1_VZV_TK, HSV-1_VZV_TK_UL29mut, and HSV-1_BAC_UL29mut strains.

(a) The recombinant HSV-1_VZV_TK strain bears a VZV TK gene in the intergenic region between the UL50 and UL51 genes, and a Kan resistance gene was used to replace the partial HSV-1 TK gene. The HSV-1_VZV_TK_clone α strain was isolated by plaque purification under ACV supplementation conditions. (b) The HSV-1_VZV_TK_UL29mut strain was constructed based on HSV-1_VZV_TK using a two-step Red-mediated mutagenesis procedure with pUC-Zeo. (c) HSV-1_BAC_UL29mut was constructed from the parental HSV-1_BAC using a two-step Red-mediated mutagenesis procedure and pcDNA3-KanS.

Table. S1 Primers used for mutagenesis

primer code	sequence (5' to 3') direction*	introduced alteration
HSV-1_VZV_TK_UL29mut F	GCGGTTGGTGATGATGGTCTCCAGCCGCCCCATGGCCGTGaGGACCGCCTGGTCCACGTACTGTAAAACGACGGCCAGTGA	P597L
HSV-1_VZV_TK_UL29mut R	GCCGAACTCGAGACCCTGCAGTACGTGGACCAGGCGGTCCtCACGGCCATGGGGCGGCTGATTACGCCAAGCTTGCATGC	
HSV-1_BAC_UL29mut F	GCGGTTGGTGATGATGGTCTCCAGCCGCCCCATGGCCGTGaGGACCGCCTGGTCCACGTACAGGATGACGACGATAAGTAGGG	P597L
HSV-1_BAC_UL29mut R	GCCGAACTCGAGACCCTGCAGTACGTGGACCAGGCGGTCCtCACGGCCATGGGGCGGCTGCAACCAATTAACCAATTCTGATTAG	
HSV-1_BAC_UL29mut_rev F	GCGGTTGGTGATGATGGTCTCCAGCCGCCCCATGGCCGTGgGGACCGCCTGGTCCACGTACAGGATGACGACGATAAGTAGGG	L597P
HSV-1_BAC_UL29mut_rev R	GCCGAACTCGAGACCCTGCAGTACGTGGACCAGGCGGTCCcCACGGCCATGGGGCGGCTGCAACCAATTAACCAATTCTGATTAG	

*Mutated and reversed sequences are shown in lower case and with underline, respectively.

Table. S2 Comparison of ACV susceptibility of all viruses in this study

cells	IC_{50} (mean ±SD μg/ml)						
	HSV-1 F strain	HSV-1_BAC	HSV-1_BAC_VZV_TK	HSV-1_BAC_VZV_TK_clone α	HSV-1_BAC_VZV_TK_UL29mut	HSV-1_BAC_UL29mut	HSV-1_BAC_UL29mut_rev
Vero cells	0.42 ± 0.07	0.41 ± 0.06	2.6 ± 0.10	7.2 ± 1.50	7.2 ± 1.70	0.65 ± 0.09	0.47 ± 0.03
HEL cells	0.27 ± 0.11	0.60 ± 0.14	NT	NT	NT	1.39 ± 0.02	0.45 ± 0.21
ARPE19 cells	23.19 ± 2.11	24.7 ± 11.0	NT	NT	NT	113 ± 1.26	27.9 ± 9.20

NT, not tested
